# Experimental and Thermodynamic Viewpoints on Claims of a Spontaneous H_2_O_2_ Formation at the Air–Water Interface

**DOI:** 10.1021/acs.jpcb.2c07394

**Published:** 2023-03-13

**Authors:** Duy Nguyen, Pin Lyu, Son C. Nguyen

**Affiliations:** Department of Chemistry and Biochemistry, University of California, Merced, California 95343, United States

## Abstract

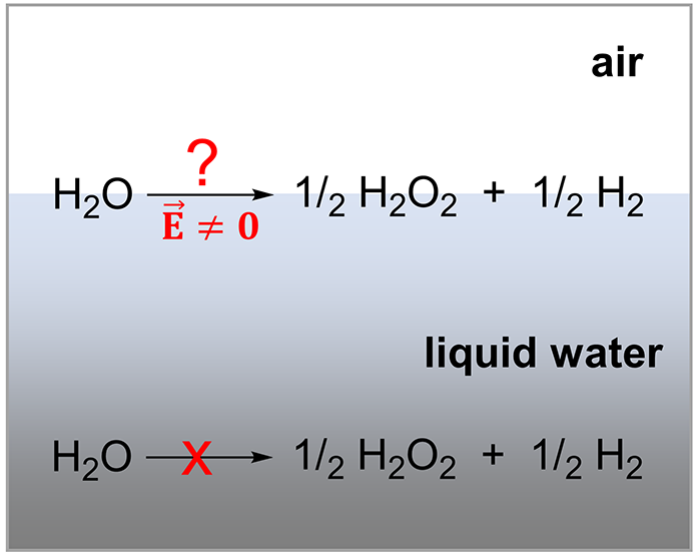

Recent claims of the spontaneous H_2_O_2_ formation at the air–water interface of water microdroplets have sparked debates on its feasibility. New results from different research groups have provided more insight into these claims, but conclusive proofs are still far from realized. In this Perspective, thermodynamic viewpoints, potential experiments, and theoretical approaches are presented as references for future studies. We suggest that future work should seek for H_2_ byproduct as indirect evidence to confirm the feasibility of this phenomenon. Examining potential energy surfaces for H_2_O_2_ formation reaction when moving from the bulk to the interface under the influence of the local electric fields is also critical to establish this phenomenon.

## 1 INTRODUCTION

Thermodynamics and kinetics of many chemical reactions at interfaces are different from those in the bulk.^1−5^ These differences come from the inhomogeneity of the media at or near the interfaces. As for the air–water interface, surrounding water imposes asymmetric molecular interactions on the observed water molecules and on solutes. The interfacial water has a lower density than bulk water, and its density fluctuations give rise to macroscopic capillary waves, surface roughness, and tension. These cause deviations in molecular dynamics, orientations, hydrogen bond networks, and dielectric properties from bulk water.^5,6^ In the case of ions or surfactants adsorbed at the air–water interface, these modify surface tension, surface potential, and eventually interfacial chemistry.^7−9^ Recently, it was reported that the surface of water microdroplets spontaneously produced H_2_O_2_, and the local electric field at the surface was claimed to be the driving force.^10,11^ These reports generated considerable attention because H_2_O_2_ formation from pure water is thermodynamically unfavorable in bulk water, and the effect of electric fields on H_2_O_2_ formation is still unsettled. It is essential to investigate these claims because the air–water interface is ubiquitous in nature and technologies. Understanding the chemistry at the air–water interface would advance our knowledge in aerosol and environmental chemistry. In this Perspective, we discuss the inconsistent experimental results from pioneering groups and lay out some potential approaches to evaluate and understand the putative H_2_O_2_ presence in the studied water microdroplets.

### 1.1 Early Reports on H_2_O_2_ Formation from Water Microdroplets

Chemical reactions at air–water interfaces have been widely studied in the context of interfacial water playing the role of a reaction solvent.^1−3,6^ However, two recent reports, here called Reports 1 and 2, claimed the H_2_O_2_ formation at the air–water interface of water microdroplets without any additives.^10,11^ This is a big surprise because thermodynamic data suggest that H_2_O_2_ formation from pure liquid water is highly unfavorable. Note that research on air–water interfaces of microdroplets still generates many disagreements.^12−14^ Taking the debate on acid–base character of surface water as an example, electrophoresis of air bubbles in water and electrospray ionization mass spectrometry of aqueous droplets suggested the excess of hydroxyl ions at the air–water interfaces.^14,15^ In contrast, sum frequency and second harmonic generation spectroscopies on flat surfaces of aqueous solutions suggested the presence or enhancement of hydronium ions at the surfaces.^16−19^ These spectroscopic results were well supported by many simulations.^20−22^ One main factor leading to these disagreements comes from different methods used to probe the chemistries at the interfaces. Claims in Reports 1 and 2 are not exempt from debate due to reproducibility, contamination, and the lack of reasonable mechanistic interpretations.^23−25^

**Scheme 1:**
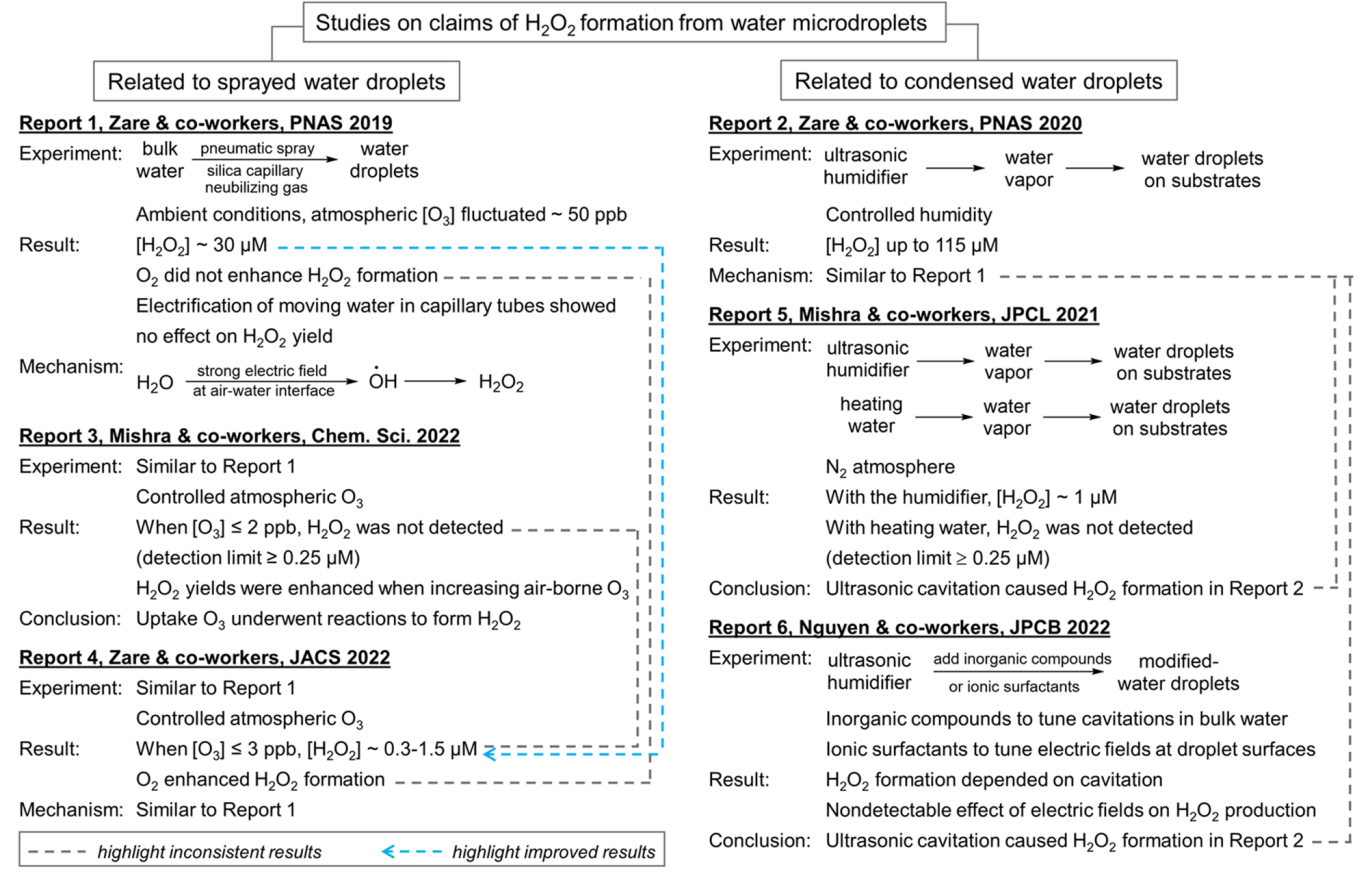
Recent Studies on the Claims of H_2_O_2_ Formation from Water Droplet Surfaces Reports 1,^10^ 2,^11^ 3,^28^ 4,^29^ 5,^30^ and 6^31^ are listed as some representative works.

In Report 1, 30 μM H_2_O_2_ was detected from water microdroplets produced via pneumatic spraying, using silica capillary tubes and N_2_ nebulizing gas (see summary in Scheme 1).^10^ Control experiments with O_2_ nebulizing gas or dissolved O_2_ in the water source did not enhance the H_2_O_2_ formation. Smaller droplets, created by increasing nebulization gas pressure, gave more H_2_O_2_. In Report 2, the water microdroplets were created by condensing water vapor on various inert substrates, and the vapor was supplied by an ultrasonic humidifier. This method of creating water droplets could avoid some undesired effects of spraying liquid, such as electrokinetics^26^ or charge separation during an aerodynamic breakup,^27^ which potentially generate H_2_O_2_. As we will point out later, using an ultrasonic humidifier may create cavitation and sonolysis in the water reservoir that generate H_2_O_2_ which later contaminates the water vapor and the studied droplets. Similar to Report 1, the amount of H_2_O_2_ in the collected droplets was quantified by titration with potassium titanium oxalate. Depending on experimental conditions, H_2_O_2_ detection could achieve in a range of 15 to 115 μM. Apparently, the reported H_2_O_2_ presence in water droplets was easily observed by unsophisticated equipment, yet it is still challenging to define the underlying reason. Considering that the air–water interface is so ubiquitous, these reported results may imply that the chemical processes creating H_2_O_2_ might have involved interfacial chemistry we have been studying. As these two reports have gained great attention, the experiments described therein were revisited with rigorous control.

### 1.2 Revisited Works with Rigorous Control and New Insight

With regard to experiments utilizing sprayed droplets, it was soon realized that H_2_O_2_ yield is very sensitive to airborne O_3_.^23−25,28^ The O_3_ could adsorb on the droplets and undergo further reactions to form H_2_O_2_. As summarized in Scheme 1, experiments in Report 1 were revisited in Reports 3 and 4. When O_3_ was scrubbed off from the gas phase to a few ppb level, Report 3 concluded that the spray droplets had no detectable H_2_O_2_ by spectrofluorometric assay^28^ (detection limit ≥0.25 μM), but Report 4 confirmed an amount of 0.3–1.5 μM by NMR and spectrofluorometric spectroscopies.^29^ Note that experiments in Report 1 did not have atmospheric O_3_ removed, and the gas phase of those air-exposed experiments could have O_3_ fluctuated around 50 ppb based on the daily data from the Environmental Protection Agency.^32^

Since Reports 3 and 4 utilized the same method to generate water droplets under rigorous controls, their inconsistent results raise concerns about reproducibility and deserve more attention. In Report 3, a careful analysis of mechanical vibrations and shock waves during pneumatic spraying ensured that the rises in local temperature and pressure were too mild to trigger a chemical transformation. Other control experiments showed that the evaporative concentration during pneumatic spray could increase the amount of contaminated H_2_O_2_ in the water source up to about 10 times in the droplets, depending on the flow rates of liquid water and the nebulizing gas. This implies that a trace of contaminated H_2_O_2_ in the water source could stay below the detection limit and pass a rigorous examination of the input, but it could later undergo evaporative concentration to reach a detectable level in the droplets. Although Report 4 used silica capillary tubes and did not confirm the effect of the capillary wall on H_2_O_2_ formation, a recent work from the same group reported that the water–silica contact actually produced H_2_O_2_.^33^ In this microfluidic setup, water flowed through channels in a silica glass substrate, and the H_2_O_2_-sensitive water-soluble probe (10-acetyl-3,7-dihydroxyphenoxazine) showed a H_2_O_2_ concentration of 56 μM. Thus, further investigation is needed to ascertain the contributions of water–solid contact, associated with evaporative concentration, to the observed H_2_O_2_ in spray experiments.

With regard to experiments utilizing condensed droplets, Reports 5 and 6 raised a concern that the water vapor source in Report 2 already had H_2_O_2_ due to ultrasonic cavitation in the used humidifier, and this H_2_O_2_ co-condensed with water vapor or adsorbed on the droplets.^30,31^ According to Report 5, when the water vapor was prepared by gently heating water liquid as a control, there was no detection of H_2_O_2_ (detection limit ≥0.25 μM).^30^ But when the vapor was prepared by an ultrasonic humidifier, the collected droplets had about 1 μM H_2_O_2_. Based on this contrast, Report 5 concluded that the humidifier, not the droplet interface, contributed to H_2_O_2_ formation. The 1 μM is smaller than the 115 μM measured in Report 2, which could be due to a larger chamber used in Report 5 that diluted the H_2_O_2_ concentration in the gas phase and resulted in less H_2_O_2_ condensing in water droplets.^31^ Report 6 used a different approach by modifying the surface of ultrasonically atomized droplets with various surfactants, but those modifications did not affect H_2_O_2_ production. The results indicate that the droplet surface does not produce H_2_O_2_. Other control experiments utilizing aqueous solutions with different gases and electrolytes confirmed that the H_2_O_2_ yield was only affected by sonochemistry in the bulk water.^31^

As we have learned from these exciting reports, the chemistry at the air–water interface is very interesting but quite challenging to study due to its sensitivity to contamination. Revisited experiments with better control conditions were very helpful to clarify these observations. Besides, reporting all experimental details was critical for reproducibility and further investigation. Although previous experiments were conducted under rigorous conditions to avoid any interference or contamination as much as possible, Report 4 confirmed H_2_O_2_ formation at the air–water interface from water droplets while Reports 3, 5, and 6 did not. Therefore, further experiments, probably with different approaches, are needed to evaluate the claims of spontaneous H_2_O_2_ formation at water droplet surfaces.

One desirable experiment is detecting H_2_ gas as the byproduct of H_2_O_2_ formation from water.^23^ Indeed, H_2_ gas is the most obvious product after balancing the self-reaction of water for generating H_2_O_2_, thus detection of H_2_ gas can indirectly prove the H_2_O_2_ formation. Furthermore, the detection of H_2_ gas can also rule out the potential contamination to the H_2_O_2_ formation, such as aforementioned O_3_. In addition to those suggested experiments, the thermodynamic aspects of the H_2_O_2_ formation also need more investigations. Moving forward, more systematic approaches are needed to tackle these claims.

## 2 MOVING FORWARD: SOME POTENTIAL NEW APPROACHES

### 2.1 Detection of H_2_O_2_ and H_2_

To evaluate the claims mentioned above, further proofs of H_2_O_2_ and H_2_ produced are still needed. Besides eliminating contamination, quantifying these species at low concentrations is also a challenging task. One way to overcome this is to accumulate enough products for detection. As we have learned from the aforementioned work, the water droplets were first formed and H_2_O_2_ was later detected from the collected droplets. These procedures actually utilized kinetic methods, wherein the product concentration was monitored after a certain reaction time. Kinetic methods have certain advantages. If the amount of H_2_O_2_ produced at the air–water interfaces is low, it can accumulate and be detected in the droplet after a sufficient time. Especially, the accumulation of H_2_ gas is more crucial for detection due to its dispersion in the gas phase. Note that H_2_ may not be produced with the presence of O_3_ or O_2_ contaminants (see eqs 3 and 4 in the Supporting Information). Furthermore, varying experimental conditions and correlating with H_2_O_2_ product yield can help with interpreting the reaction mechanism, such as changing droplet size to evaluate the effect of the droplet curvature or changing experiment temperatures to estimate the reaction activation energy.

Another approach is preparing droplets with probing chemicals such that their reactions indicate the existence of H_2_O_2_^10,34^ or intermediates in H_2_O_2_ formation, e.g., hydroxyl radicals.^35^ This method not only indirectly proves H_2_O_2_ formation but also demonstrates the influence of H_2_O_2_ and hydroxyl radicals on other reactions.

The kinetic method, however, has its drawbacks, such as not directly detecting reaction intermediates nor probing properties of air–water interfaces. Some interface-sensitive spectroscopies, such as sum-frequency generation (SFG),^36^ can potentially probe intermediates or H_2_O_2_ product at water droplet surfaces. Recent glancing-angle Raman spectroscopy on 1 M H_2_O_2_ solution confirmed the surface propensity of H_2_O_2_ at the water–air interface with the standard free energy adsorption of −1.2 kcal/mol.^37^ This adsorption energy had also been predicted by molecular dynamics (MD) simulations.^38,39^ However, low concentrations of these species could be a challenge for detection.

### 2.2 Thermodynamic Considerations of H_2_O_2_ Formation from Water Droplets

The claims of spontaneous H_2_O_2_ formation from water droplets invite thermodynamic considerations. A table of thermodynamic quantities of chemical species that could be relevant to this reaction is provided in the Supporting Information for any future investigations. As H_2_ is the expected byproduct, we use the H_2_O_(l)_ → 1/2 H_2_O_2(aq)_ + 1/2 H_2(g)_ reaction to establish our thermodynamic viewpoints. Starting from thermodynamic data, this reaction in the bulk has a standard Gibbs free energy (*ΔG*_bulk rxn_^o^) of 40.7 kcal/mol (see the calculation in the Supporting Information), and it does not spontaneously occur. The same reaction in the gas phase has a *ΔG*^o^ of 42.1 kcal/mol. Note that the reactions between water and O_2_ or O_3_ to form H_2_O_2_ in liquid water have the *ΔG*_rxn_^o^ of 24.6 or −14.4 kcal/mol, respectively (see eq 3 and 4 in the Supporting Information). These values are significantly less positive, or even become negative, as compared to the 40.7 kcal/mol of the water self-reaction mentioned above. Thus, O_2_ and O_3_ contaminants must be eliminated from future studies.

**Figure 1:**
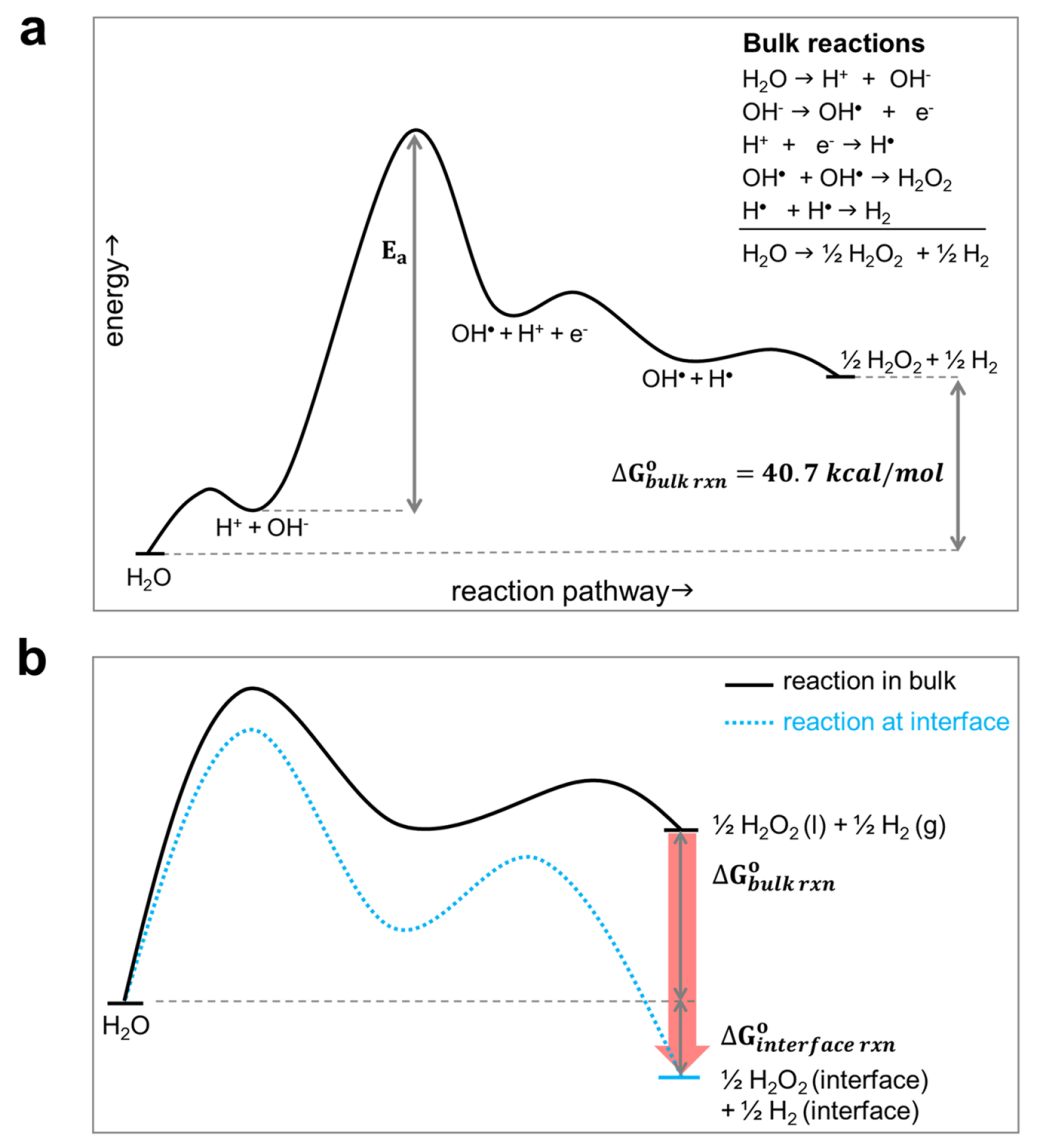
(a) A possible pathway to demonstrate the uphill H_2_O_2_ formation from water in the bulk. (b) To make this reaction happen spontaneously at the air–water interface, the reaction pathway must be shifted to favor the products when moving from water bulk to surface. Current thermodynamic data do not support this energy shift. The red arrow indicates possible shifts in energy levels when moving from bulk to the interface.

Figure 1a illustrates an educated-guess pathway to demonstrate the endothermic reaction of water into H_2_O_2_ and H_2_ in solution. In order for this reaction to happen spontaneously at the air–water interface, the reaction potential energy surface (PES) must shift in favor of the products (i.e., *ΔG*_rxn at interface_^o^ < 0). In other words, when moving from bulk to interfaces, the energy levels of the reactant and products must be changed. Although the reaction pathway is still not known in detail and is not the focus of our thermodynamic standpoint, the emphasis in Figure 1 is the relative Gibbs free energy of the reactant and products. The Gibbs free energy of H_2_O must be unchanged when water transfers between the bulk and interface because the initial and final systems are equivalent. Hence, the only possibility that could explain the spontaneous H_2_O_2_ formation from water droplets is the Gibbs free energies of H_2_O_2_ and H_2_ decrease when moving from bulk to the interface (Figure 1b). H_2_O_2_ is energetically −1.2 kcal/mol more favorable at the air–water interface (as compared to its free energy in liquid water) as measured recently by glancing-angle Raman spectroscopy.^37^ MD simulations showed that H_2_O_2_ and other small molecule gases such as N_2_ and O_2_ are about −1 kcal/mol more favorable at the air–water interface than in water.^38^ H_2_ is expected to have an energy profile with a less noticeable change. Combing these thermodynamic data, the reaction of H_2_O_(l)_ → 1/2 H_2_O_2(aq)_ + 1/2 H_2(g)_ at the air–water interface is expected to have lower free energy of the products by roughly about −1 kcal/mol as compared to the same reaction in the bulk. This energy shift is unlikely to overcome the 40.7 kcal/mol mentioned above.

### 2.3 Considerations of the Proposed Mechanism of H_2_O_2_ Formation from Water Droplets

Understanding the mechanism of H_2_O_2_ formation from water microdroplets is probably the most challenging task. It is difficult to find a straightforward molecular interpretation for dramatically shifting the reaction pathway illustrated in Figure 1b. Reports 1 and 2 propose that the local electric field at the air–water interface is strong enough to ionize hydroxide ions (OH^–^) into hydroxyl radicals (**^•^**OH), and the radicals then can combine to form H_2_O_2_. Report 1 also suggests that the reduction potential of the H_2_O_2_,H^+^/H_2_O couple could be lower at the interface than in the bulk due to the interface/bulk difference in solvation energy.^10,40^ This proposed **^•^**OH pathway provides a very good starting point for further mechanistic studies because OH^–^ and **^•^**OH are probably the best guesses for the starting material and intermediate, respectively. The vertical ionization energies (VIEs) of OH^–^ are much smaller than those values of H_2_O for both gas and liquid phases (see ionization energies in Table S1).^41^ Note that the VIEs of OH^–^ and H_2_O at the water microdroplet surface are still unknown, but we can expect that they follow the same trend as in gas and bulk phases. MD simulations for 4 nm water droplets show that the VIE distribution of surface OH^–^ is bimodal. One major peak is close to the experimental VIEs in the bulk, and the extra peak is about a hundred kcal/mol lower.^41^ Hence, the water-ionization pathway for H_2_O_2_ formation is unlikely to happen.

The effect of the electric field on the PES of a reaction has recently gained attention, mostly in the context of reducing the activation energy, but not so much about changing *ΔG* of a reaction. For enzyme catalysis, it is proposed that the active sites can be electrostatically preorganized to stabilize the transition states of the catalyzed reactions and effectively reduce the reaction activation energy.^42^ For example, the wild-type ketosteroid isomerase can exert an electric field of 144 MV/cm on the C=O bond involved in the transition state.^42^ Designing local electric fields to shift the PES and improve catalytic activity or selectivity will be a new toolbox in chemical synthesis.^43−45^ However, to validate claims of H_2_O_2_ formation in Reports 1 and 2, the two key questions needed to be addressed are, (i) how strong is the local electric field at the water droplet surface and (ii) can this electric field, potentially in combination with other surface effects (mentioned in the Introduction), shift the reaction PES to favor H_2_O_2_ product at the interface?

#### 2.3.1 How Strong Is the Electric Field at the Air–Water Interfaces of Water Droplets?

At a certain time and location at the interfaces, an interfacial water molecule must experience a local electric field induced by the neighbor molecules. However, it is still quite challenging to probe this electric field at the air–water interfaces of the droplets by experiments. Recently, an electric field of around 10 MV/cm at the oil–water interface of aqueous microdroplets was observed using a nitrile-bearing fluorescent probe and stimulated Raman excited fluorescence microscopy.^46^ However, adding spectators to probe the electric field strength by Stark effects may not be a good option, as they may alter the original electric field of the pristine air–water interface. Sum frequency generation spectroscopy (SFG) on the flat and clean air–water interface provided information about the local environment at the interface,^47−49^ and the observed spectral shift of the OH stretching was assigned to different types of hydrogen bonding of interfacial water.^50^ Noticeably, the dangling OH bond pointing toward the vapor phase has a frequency of ∼3700 cm^–1^. This frequency can report the local electric field at the air–water interface. Since the experimental Stark tuning rate, the frequency shift in response to the projected electric field along the observed chemical bond, is not yet available for the OH dangling bond vibration, the electric field cannot be determined directly from this experimental frequency.

However, MD simulation using the extended simple point charge model can help us estimate this electric field. Basically, this model establishes an empirical correlation between the observed vibrational frequencies of the OH stretching modes of water and the calculated electric field exerted at the H atom and projected along the OH bond.^51−54^ This electric field was summed up from the electric field of atoms from neighboring-water molecules. This correlation, also known as the OH frequency map, is quite robust and can be applied to bulk, surface, and cluster water.^54^ Using Figure 2 in ref (54), the corresponding electric field for the 3700 cm^–1^ vibration is about 0.01 atomic units or 50 MV/cm. SFG spectroscopy on a flat water surface also detected a strong intensity in the 3400–3100 cm^–1^ region, which was assigned to the signal of water molecules residing next to the adjacent surface water molecules.^50^ Using the same empirical correlation, this low frequency region corresponds to an electric field of about 200–260 MV/cm. Note that this estimation is extrapolated from measurements on flat surfaces, and we are trying to apply it to micron-sized water droplets mentioned in Sections 1.1 and 1.2.

Recent MD simulations for water droplets of 8–16 nm in diameter show that the electric field at the droplet surface exhibits a Lorentzian distribution in which its center value is less than 9 MV/cm but its tail can reach to hundreds of MV/cm.^55^ These electric fields were calculated at specific points on the droplet surface, and their strength is not far from the values obtained from the above vibrational frequency map. These results indicate that the water molecules at or near the interfaces can have some thermally fluctuating arrangements that randomly produce a very high local electric field, possibly up to several hundreds of MV/cm.

Even though a local electric field up to several hundreds of MV/cm at the air–water interface seems to be a high value, it is important to know that this field strength is not surprisingly high when compared to the bulk value. The continuum solvent model estimates that the surrounding water can exert an electric field up to about 200 MV/cm on a water molecule in the bulk.^56^ As liquid water has the OH stretching frequency in the 3700–2800 cm^–1^ region, the corresponding electric field estimated from the frequency map method is about 0 to 300 MV/cm.^54^ Apparently, these strong local electric fields do not cause H_2_O_2_ formation in bulk water.

#### 2.3.2 Can a Strong Electric Field Shift the PES of the Water Reaction to Favor H_2_O_2_ Product at the Interface?

**Figure 2:**
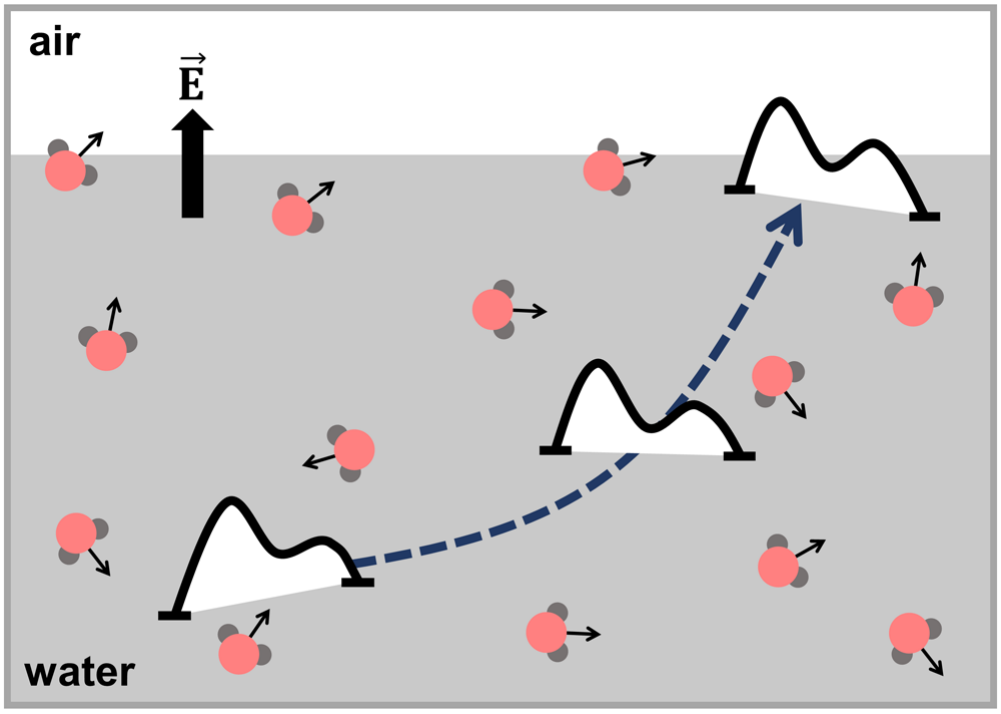
In order to investigate whether the H_2_O_(l)_ → 1/2 H_2_O_2(aq)_ + 1/2 H_2(g)_ reaction spontaneously occurs at the water droplet surface, the reaction pathway should be determined and projected from the bulk to the interface. The effect of local electric fields on the pathway should also be considered.

We anticipate that electric fields can generally influence thermodynamics and kinetics of a chemical reaction, but it is still unknown at which strength they can shift the PES of this reaction. The same MD simulations for 8–16 nm water droplets mentioned above show that the projected electric field on the OH bonds of water molecules inside the droplets has a distribution centered at 0.3 MV/cm and that its width is about tens of MV/cm.^55^ Note that this electric field strength is different from values obtained from the frequency map method^54^ and the continuum solvent model^56^ mentioned above. This difference comes from where and how the local electric field was calculated. The same MD simulations for the free OH bonds of surface water have a broad distribution centered at ∼16 MV/cm, and this field strength can destabilize the OH bond.^55^ The tail of this distribution also can reach up to about a hundred MV/cm. While it was not clearly confirmed that this destabilization could be sufficient to shift the PES to favor the H_2_O_2_ product, those results suggest that the large local electric field could be a source of H_2_O_2_ formation. However, future work must perform a full calculation of the PES when moving from bulk to interfacial water under the effect of local electric fields. Figure 2 illustrates this approach by depicting the shift of the reaction pathway.

A recent study focuses on the ionization energy of OH^–^ forming **˙**OH at the air–water interface, wherein the VIEs of partially solvated OH^–^ ions are greatly lowered relative to the average VIE of a fully solvated OH^–^ in the liquid phase.^41^ Although this MD simulation provides an important explanation for the possible formation of OH**˙** due to electric field fluctuation at the droplet surfaces, a key step in H_2_O_2_ formation, this result is for 4 nm water droplets and quite far from a complete picture of the PES.

## 3 OUTLOOK

Current research on claims of spontaneous H_2_O_2_ formation at the air–water interface of water droplets ultimately reflects our limited understanding of interfacial chemistry. With the quick response from our research community, new results with better control and consideration have provided more insight, but conclusions on the feasibility of these claims remain unsettled. Reporting experimental details has been critical to reproducibility and self-correction. Given their significant impacts, these claims deserve further experimental confirmations and theoretical interpretation, such as detecting the H_2_ byproduct and determining the Gibbs free energy of this interfacial reaction. We hope that the brief discussions on experimental and thermodynamic approaches presented here will inspire more researchers to participate in this intriguing research direction.

## Supporting Information Available

Standard reaction Gibbs free energies and table of thermodynamic quantities (PDF)

## ABBREVIATIONS

NMR: nuclear magnetic resonance
SFG: sum-frequency generation
PES: potential energy surface
VIE: vertical ionization energy
MD: molecular dynamics
