# Supplementary material for: Experimental and Thermodynamic Viewpoints on Claims of a Spontaneous H2O2 Formation at the Air–Water Interface
Source: J Phys Chem B. 2023 Mar 13;127(11):2323–30. doi: 10.1021/acs.jpcb.2c07394 (PMC10041628; doi:10.1021/acs.jpcb.2c07394)
Supplement: Supplementary file 1 — jp2c07394_si_001.pdf [file jp2c07394_si_001.pdf]

Supporting information for "Experimental and Thermodynamic Viewpoints on Claims of a Spontaneous H_2O_2 Formation at the Air-Water Interface"

*Duy Nguyen, Pin Lyu, and Son C. Nguyen**

Department of Chemistry and Biochemistry, University of California, Merced, CA 95343, United States

Corresponding Author: son@ucmerced.edu

1. Standard Gibbs free energies of some reactions for H_2O_2 formation in water solution and gas phase.

$H_2O_{(l)} \rightarrow \frac{1}{2} H_2O_{2(aq)} + \frac{1}{2} H_{2(g)}$ (1)

$$\Delta G^\circ = \frac{1}{2}\left(\Delta G^\circ_{H_2O_2,aq} + \Delta G^\circ_{H_2,g}\right) - \Delta G^\circ_{H_2O,l} = \frac{1}{2}\left(-134.1\ \mathrm{kJ\cdot mol^{-1}} + 0\ \mathrm{kJ\cdot mol^{-1}}\right) - \left(-237.1\ \mathrm{kJ\cdot mol^{-1}}\right) = 170.0\ \mathrm{kJ\cdot mol^{-1}} = 40.7\ \mathrm{kcal\cdot mol^{-1}}$$

$H_2O_{(g)} \rightarrow \frac{1}{2} H_2O_{2(g)} + \frac{1}{2} H_{2(g)}$ (2)

$$\Delta G^\circ = \frac{1}{2}\left(\Delta G^\circ_{H_2O_2,g} + \Delta G^\circ_{H_2,g}\right) - \Delta G^\circ_{H_2O,g} = \frac{1}{2}\left(-105.6\ \mathrm{kJ\cdot mol^{-1}} + 0\ \mathrm{kJ\cdot mol^{-1}}\right) - \left(-228.6\ \mathrm{kJ\cdot mol^{-1}}\right) = 175.8\ \mathrm{kJ\cdot mol^{-1}} = 42.1\ \mathrm{kcal\cdot mol^{-1}}$$

$H_2O_{(l)} + \frac{1}{2} O_{2(g)} \rightarrow H_2O_{2(aq)}$ (3)

$$\Delta G^\circ = \Delta G^\circ_{H_2O_2,aq} - \left(\Delta G^\circ_{H_2O,l} + \frac{1}{2}\Delta G^\circ_{O_2,g}\right) = -134.1\ \mathrm{kJ\cdot mol^{-1}} - \left(-237.1\ \mathrm{kJ\cdot mol^{-1}} + \frac{1}{2}\times 0\ \mathrm{kJ\cdot mol^{-1}}\right) = 103.0\ \mathrm{kJ\cdot mol^{-1}} = 24.6\ \mathrm{kcal\cdot mol^{-1}}$$

$H_2O_{(l)} + O_{3(g)} \rightarrow H_2O_{2(aq)} + O_{2(g)}$ (4)

$$\Delta G^\circ = \left(\Delta G^\circ_{H_2O_2,aq} + \Delta G^\circ_{O_{2,g}}\right) - \left(\Delta G^\circ_{H_2O,l} + \frac{1}{2}\Delta G^\circ_{O_{3,g}}\right) = \left(-134.1\ \mathrm{kJ\cdot mol^{-1}} + 0\ \mathrm{kJ\cdot mol^{-1}}\right) - \left(\left(-237.\,1\ \mathrm{kJ\cdot mol^{-1}}\right) + 163.2\ \mathrm{kJ\cdot mol^{-1}}\right) = -60.2\ \mathrm{kJ\cdot mol^{-1}} = -14.4\ \mathrm{kcal\cdot mol^{-1}}$$

All the above data were extracted from Ref. 1

2. Thermodynamic quantities of chemical species that may be relevant to H_2O_2 formation

Table S1. Thermodynamic quantities of chemical species that could be relevant to hydrogen peroxide formation

Chemical species		H_2O	•OH	H_2O_2	H_2	H_3O^+	OH^-	e^-
Enthalpy of Formation (ΔH_f°, kJ·mol^{-1})	Gas phase	-241.8 [1]	39.0 [1]	-136.3 [1]	0	584.2 [2]	-143.6 [2]	
	Interface							
	Bulk	-285.83 (l) [1]	-0.8 ± 5.9 (aq) [3]	-187.8 (l) [1]<br>-191.2 (aq) [1]		144.3 (aq) [4]	-230.0 (aq) [1]	277.4 (aq) [5]
Gibbs Energy of Formation (ΔG_f°, kJ·mol^{-1})	Gas phase	-228.6 [1]	34.2 [1]	-105.6 [1]	0	606.6 [2]	-138.7 [2]	
	Interface							
	Bulk	-237.1 (l) [1]	24.3 (aq) [3]	-120.4 (l)[1]<br>-134.1 (aq)[1]		183.9 (aq) [4]	-157.3 (aq) [1]	276.9 (aq) [5]
Entropy (S°, J·K^{-1}·mol^{-1})	Gas phase	188.8 [1]	183.6 [1]	232.7 [1]	130.7 [1]	192.3 [2]	172.5 [2]	
	Interface							
	Bulk	69.9 [1]		109.6 (l) [1]<br>143.9 (aq) [1]			-10.9 (aq) [1]	66.9 (aq) [5]
Solvation Energy* (kJ·mol^{-1})	Gas phase							
	Interface		-17.3 (cal) [6]<br>-16.74 (cal) [7]	-43.9 (cal) [8]		-424.7 (cal) [9]	-440.6 (stabilized by 1 k_bT) [10]	-154.4 (VBE, exp) [11]
	Bulk	-29.8 (aq, cal) [12]<br>-26.4 (aq, exp) [13]	-13.4 (aq, cal) [6]<br>-12.5 (l, cal) [7]<br>-16.32 (l, exp) [7]	-41.8 (aq, cal) [8]<br>-36.6 (aq, exp) [8]	9.2 (cal) [14]<br>9.79 (exp) [15]	651.0 (aq, con, cal) [16]<br>-461.5 (aq, abs, cal) [16]<br>-439.3 (aq, cal) [9]	-1550.6 (aq, con, cal) [16]<br>-438.1 (aq, abs, cal) [16]	-164.8 (exp) [17]<br>-148.5 (cal) [18]<br>-318.4 (VBE, exp) [11]
Henry's Law Constants (H_s^{cp}, mol^{-1}·m^{-3}·Pa^{-1})	Water solvent		0.38 [19]	910 [19]	7.80×10^{-6} [19]			

Ionization Energy (eV)	Gas phase	12.61 (exp)[1]	13.02 (cal)[20] 13.02 (exp)[21]	10.64 (cal)[22] 10.65 (exp)[22]	15.32 (cal)[23] 15.43 (exp)[1]		1.90 (VDE, exp)[24]	
	Interface	10.30 (cal)[25]						
	Bulk	~10 (exp)[25] 10.55 (cal)[25]		10.54 (aq, exp)[26]		14.5 (cal)[27]	9.20 (exp)[27] 9.70 (cal)[27]	3.3 (VBE, cal)[28] 3.3-3.7 (VBE, exp)[29]
Standard Reduction Potential (V)	Gas phase							
	Interface							
	Bulk	-0.8 (H_2O/H_2, OH^-)[1]	2.7 (·OH, H^+/H_2O)[30] 1.9 (·OH/OH^-)[30]	0.8 (H_2O_2, H^+/H_2O,·OH)[30] 1.8 (H_2O_2, H^+/H_2O)[1]				-2.7 (aq/e_{aq}^-)[30]

*The absolute and conventional solvation free energies are presented. The absolute value of a single ion cannot be measured, the conventional value is therefore often tabulated by arbitrarily setting the free energy of solvation of the proton equal to zero.[16]

Note. water solvent unless specified, l – liquid, aq – aqueous, VBE – vertical binding energy, VDE – vertical detachment energy, abs – absolute, cal – calculated.

References

(1) James Speight, P. D. *Lange's Handbook of Chemistry, Sixteenth Edition*. 16th / ed.; McGraw-Hill Education: New York, 2005.

(2) Chase, M. NIST-JANAF Thermochemical Tables, 4th Edition. American Institute of Physics, -1: 1998.

(3) Autrey, T.; Brown, A. K.; Camaioni, D. M.; Dupuis, M.; Foster, N. S.; Getty, A. Thermochemistry of Aqueous Hydroxyl Radical from Advances in Photoacoustic Calorimetry and ab Initio Continuum Solvation Theory. *J. Am. Chem. Soc.* **2004,** *126,* 3680-3681.

(4) Nikolai, B.; Manoj, K. M. Thermodynamics of Hydronium, Zundel and Eigen ions. *OSF Preprints* **2021,** *doi:10.31219/osf.io/b6v24*.

(5) Schwarz, H. A. Enthalpy and Entropy of Formation of the Hydrated Electron. *J. Phys. Chem.* **1991,** *95,* 6697-6701.

(6) Wick, C. D.; Dang, L. X. Computational Observation of Enhanced Solvation of the Hydroxyl Radical with Increased NaCl Concentration. *J. Phys. Chem. B* **2006,** *110,* 8917-8920.

(7) Roeselová, M.; Vieceli, J.; Dang, L. X.; Garrett, B. C.; Tobias, D. J. Hydroxyl Radical at the Air−Water Interface. *J. Am. Chem. Soc.* **2004,** *126,* 16308-16309.

(8) Vácha, R.; Slavíček, P.; Mucha, M.; Finlayson-Pitts, B. J.; Jungwirth, P. Adsorption of Atmospherically Relevant Gases at the Air/Water Interface: Free Energy Profiles of Aqueous Solvation of N_2, O_2, O_3, OH, H_2O, HO_2, and H_2O_2. *J. Phys. Chem. A* **2004,** *108,* 11573-11579.

(9) Dang, L. X. Solvation of the Hydronium Ion at the Water Liquid/Vapor Interface. *J. Chem. Phys.* **2003,** *119,* 6351-6353.

(10) Mundy, C. J.; Kuo, I. F. W.; Tuckerman, M. E.; Lee, H.-S.; Tobias, D. J. Hydroxide Anion at the Air–Water Interface. *Chem. Phys. Lett.* **2009,** *481,* 2-8.

(11) Siefermann, K. R.; Liu, Y.; Lugovoy, E.; Link, O.; Faubel, M.; Buck, U.; Winter, B.; Abel, B. Binding Energies, Lifetimes and Implications of Bulk and Interface Solvated Electrons in Water. *Nat. Chem.* **2010,** *2,* 274-279.

(12) Feldt, J.; Miranda, S.; Pratas, F.; Roma, N.; Tomás, P.; Mata, R. A. Optimization and Benchmarking of a Perturbative Metropolis Monte Carlo Quantum Mechanics/Molecular Mechanics Program. *J. Chem. Phys.* **2017,** *147,* 244105.

(13) Ben-Naim, A.; Marcus, Y. Solvation Thermodynamics of Nonionic Solutes. *J. Chem. Phys.* **1984,** *81,* 2016-2027.

(14) Sabo, D.; Varma, S.; Martin, M. G.; Rempe, S. B. Studies of the Thermodynamic Properties of Hydrogen Gas in Bulk Water. *J. Phys. Chem. B* **2008,** *112,* 867-876.

(15) Young, C. L. *Hydrogen and Deuterium*. Pergamon: Oxford, England, 1981.

(16) Kelly, C. P.; Cramer, C. J.; Truhlar, D. G. Aqueous Solvation Free Energies of Ions and Ion−Water Clusters Based on an Accurate Value for the Absolute Aqueous Solvation Free Energy of the Proton. *J. Phys. Chem. B* **2006,** *110,* 16066-16081.

(17) Noyes, R. M. Assignment of Individual Ionic Contributions to Properties of Aqueous Ions. *J. Am. Chem. Soc.* **1964,** *86,* 971-979.

(18) Zhan, C.-G.; Dixon, D. A. The Nature and Absolute Hydration Free Energy of the Solvated Electron in Water. *J. Phys. Chem. B* **2003,** *107,* 4403-4417.

(19) Sander, R. Compilation of Henry's Law Constants (version 4.0) for Water as Solvent. *Atmos. Chem. Phys.* **2015,** *15,* 4399-4981.

(20) Ruscic, B.; Wagner, A. F.; Harding, L. B.; Asher, R. L.; Feller, D.; Dixon, D. A.; Peterson, K. A.; Song, Y.; Qian, X.; Ng, C.-Y.; Liu, J.; Chen, W.; Schwenke, D. W. On the Enthalpy of Formation of Hydroxyl Radical and Gas-Phase Bond Dissociation Energies of Water and Hydroxyl. *J. Phys. Chem. A* **2002,** *106,* 2727-2747.

(21) Wiedmann, R. T.; Tonkyn, R. G.; White, M. G.; Wang, K.; McKoy, V. Rotationally Resolved Threshold Photoelectron Spectra of OH and OD. *J. Chem. Phys.* **1992,** *97,* 768-772.

(22) Changala, P. B.; Nguyen, T. L.; Baraban, J. H.; Ellison, G. B.; Stanton, J. F.; Bross, D. H.; Ruscic, B. Active Thermochemical Tables: The Adiabatic Ionization Energy of Hydrogen Peroxide. *J. Phys. Chem. A* **2017,** *121,* 8799-8806.

(23) III, R. D. J. NIST Computational Chemistry Comparison and Benchmark Database. Release 22, May 2022, ed.; The National Institute of Standards and Technology (NIST) NIST Standard Reference Database Number 101, 2022; p DOI:10.18434/T47C7Z. http://cccbdb.nist.gov/.
(24) Kang, M.; Park, C. J.; Kim, H. M.; Lee, S. H. Photoelectron Spectroscopy of OH^{-}-Anion–Water Clusters Generated by Ultrasonic Nebulizer. *Int. J. Mol. Sci.* **2022,** *23,* 4175.
(25) Gaiduk, A. P.; Pham, T. A.; Govoni, M.; Paesani, F.; Galli, G. Electron Affinity of Liquid Water. *Nat. Commun.* **2018,** *9,* 247.
(26) Brown, R. S. A Photoelectron Investigation of the Peroxide Bond. *Can. J. Chem.* **1975,** *53,* 3439-3447.
(27) Winter, B.; Faubel, M.; Hertel, I. V.; Pettenkofer, C.; Bradforth, S. E.; Jagoda-Cwiklik, B.; Cwiklik, L.; Jungwirth, P. Electron Binding Energies of Hydrated H_3O^+ and OH^-: Photoelectron Spectroscopy of Aqueous Acid and Base Solutions Combined with Electronic Structure Calculations. *J. Am. Chem. Soc.* **2006,** *128,* 3864-3865.
(28) Coe, J. V.; Lee, G. H.; Eaton, J. G.; Arnold, S. T.; Sarkas, H. W.; Bowen, K. H.; Ludewigt, C.; Haberland, H.; Worsnop, D. R. Photoelectron Spectroscopy of Hydrated Electron Cluster Anions, $(H_2O)^{-}_{n=2-69}$ *J. Chem. Phys.* **1990,** *92,* 3980-3982.
(29) Nishitani, J.; Yamamoto, Y.-i.; West, C. W.; Karashima, S.; Suzuki, T. Binding Energy of Solvated Electrons and Retrieval of True UV Photoelectron Spectra of Liquids. *Sci. Adv.* **2019,** *5,* eaaw6896.
(30) Wardman, P. Reduction Potentials of One-Electron Couples Involving Free Radicals in Aqueous Solution. *J. Phys. Chem. Ref. Data* **1989,** *18,* 1637-1755.
